# JW-1-283 inhibits melanoma tumor growth via stabilization of the p53 pathway

**DOI:** 10.1016/j.gendis.2023.06.009

**Published:** 2023-07-17

**Authors:** Yang Xie, Ruida Hou, Kelli L. Hartman, Jianxiong Jiang, Zhongzhi Wu, Wei Li

**Affiliations:** Drug Discovery Center, College of Pharmacy, University of Tennessee Health Science Center, Memphis, TN 38163, USA

Melanoma is a lethal skin malignancy and the fifth most diagnosed cancer in the United States.^1^ Currently, for unresectable melanoma, the recommended treatment options include checkpoint inhibitor immunotherapy targeting programmed cell death 1 (PD-1), and inhibitors targeting the mitogen-activated protein kinase (MAPK) pathway.^2^ However, the response rate may vary among the patients, and therefore demanding the development of novel treatment strategies. Tumor suppressor TP53 has been suggested as one of the critical regulators mediating the aggressiveness and progression of melanoma. Over 80% of melanoma patients harbor the wild type (WT) yet malfunctional p53, which leads to the investigation that focused on restoring the function of p53 in melanoma as an alternative treatment strategy.^3^ The degradation of p53 is catalyzed by mouse double minute 2 (MDM2), a well-known E3 ubiquitin ligase. Based on a previously reported MDM2 inhibitor, MX25, which has an iodide counter ion and is highly susceptible to oxidation in the air, we designed a new MX25 analog JW-1-283 using a stable ethyl sulfate group as the counter ion. In the current research, we investigated the anti-melanoma effect of JW-1-283 and leverages it as a novel p53-activating reagent.

We developed compound JW-1-283 with improved biological and chemical properties (Supplementary Scheme S1). To start with, cytotoxicity of JW-1-283 against melanoma cell lines with different p53 status was determined by MTS assay with A375 (WT p53), M14 (impaired p53 functional mutation), and RPMI7951 (TP53 non-expression) human melanoma cell lines. JW-1-283 exhibited the highest potency against WT p53 A375 melanoma cell line, with an IC_50_ of 2.2 μM. For M14 and RPMI-7951, the IC_50_ values were 11.3 μM and 11.1 μM, respectively (Fig. 1A). To further investigate the effect of JW-1-283 on melanoma cell growth, A375 and M14 cell lines that represent two different p53 status were chosen for the colony formation and sphere formation assay. In the colony formation assay, JW-1-283 treatment could significantly reduce the colonies derived from A375 cells up to 91% (Fig. 1B; Fig. S1A). Although the colonies of M14 cells were also suppressed by JW-1-283, the magnitude of reduction was less salient when compared with A375 cells (Fig. 1B; Fig. S1A). Similarly, in the sphere formation assay, JW-1-283 inhibited the spheroid sizes of A375 cells dose dependently (Fig. S1B, C). Again, the magnitude of sphere size shrinkage for M14 cells was not as salient as in the case of A375 (Fig. S1B, C). Taken together, these results may imply that JW-1-283 could inhibit the proliferation of melanoma cells, with a preference of WT p53.

**Figure 1:**
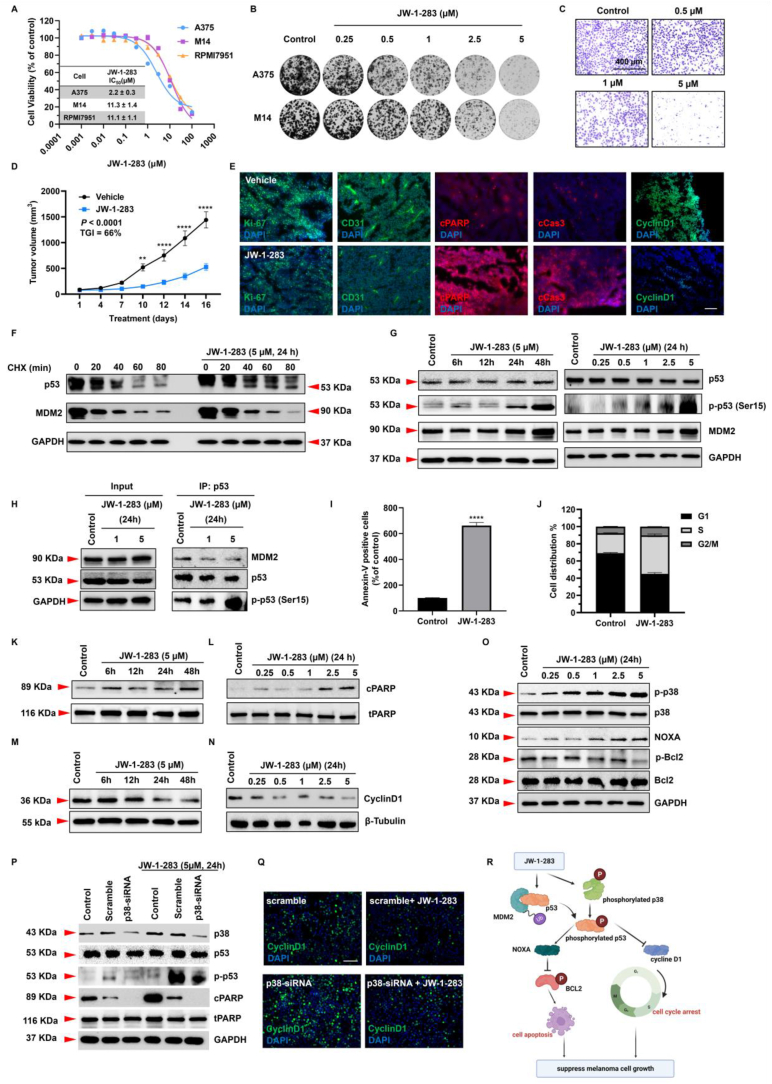
JW-1-283 inhibits melanoma tumor growth via stabilization of the p53 pathway. **(A)** The antiproliferative effect of JW-1-283 determined by MTS assay after 72 h in three melanoma cell lines with different p53 status (A375: wild-type; M14: impaired p53 functional mutation; RPMI-7951: p53 non-expression). IC_50_ values of each melanoma cell lines treated by indicated concentrations of JW-1-283. **(B)** Representative images of cell colony formation assays using A375 and M14 cells, treating with increasing concentrations of JW-1-283. **(C)** Directed migration of A375 cells was determined using a transwell 24-well plate following treatment with JW-1-283 from 0.5 to 5 μM at 24 h using crystal violet staining. Scale bar = 400 μm. **(D)** A375 cells were subcutaneously injected into the dorsal flank of NSG mice. When average tumor volume reached around 100 mm^3^, mice were randomized into 2 groups (*n* = 7/group) and treated with vehicle control (saline) or with 7.5 mg/kg doses of JW-1-283 (every other day, i. p.). The tumor growth curve indicated by tumor volume monitored every 2–3 days till the end of the treatment. The TGI was calculated to indicate the overall tumor growth inhibitory effect. **(E)** Immunostaining for Ki-67 (green fluorescence) was performed to identify proliferating cells from mice treated by vehicle and JW-1-283; CD31 (PECAM-1, green fluorescence) was utilized to indicate the vasculature formation; cleaved-PARP (cPARP) and cleaved caspase-3 (cCas3) (red fluorescence) were performed to indicate apoptosis; Cyclin D1 (green fluorescence) was used to indicate the G1/S phase cell cycle arrest. Nuclei within each tumor were counterstained with DAPI (blue fluorescence). Scale bar = 50 μm. **(F)** A375 melanoma cells treated for 24 h with 5 μM of JW-1-283 or solvent followed by cycloheximide treatment from 0 to 80 min (CHX; 100 μg/ml). Immunoblotting analysis was performed for p53 and MDM2. GAPDH as a housekeeping gene was used as a loading control. **(G)** A375 cells were either treated with 5 μM of JW-1-283 at indicated time course from 0 to 48 h or treated with indicated concentrations from 0.25 to 5 μM of JW-1-283. Immunoblotting analysis was performed for p53, p-p53 and MDM2. GAPDH as a loading control. **(H)** Coimmunoprecipitation (Co-IP) was performed in A375 cells treated with 1 and 5 μM of JW-1-283 for 24 h. Immunoblotting analysis was performed for MDM2, p53, p-p53. Input is the whole cell lysate and GAPDH was used as a loading control in input samples. p53 in IP group was used as loading control of IP samples. **(I)** Flow cytometry analysis of apoptotic cells were detected by Annexin-V/PI staining in A375 cells. Quantification of the percentage of the apoptotic cells in A375 cells treated with 5 μM of JW-1-283 at 24 h, comparing with vehicle control of no treatment. **(J)** A375 cells were treated with 5 μM of JW-1-283 after 24 h. Cell-cycle distribution was determined by flow cytometry. The columns indicate the percentage of cells in each phase of the cell cycle. Data is shown as the grand mean ± SEM calculated from three independent experiments. (G1/S phase, *P* < 0.0001) **(K)** and **(L)** A375 cells were either treated with 5 μM of JW-1-283 at indicated time course from 0 to 48 h or treated with indicated concentrations from 0.25 to 5 μM of JW-1-283. Immunoblotting analysis was performed for apoptotic marker total and cPARP. **(M)** and **(N)** A375 cells were either treated with 5 μM of JW-1-283 at indicated time course from 0 to 48 h or treated with indicated concentrations from 0.25 to 5 μM of JW-1-283. Immunoblotting analysis was performed for G1/S phase marker Cyclin D1. β-Tubulin was adopted as loading controls. **(O)** A375 cells were treated with indicated concentrations from 0.25 to 5 μM of JW-1-283. Cells were lysed after 24 h treatment and immunoblotting analysis was performed for p53, p-p53, p38, phosphorylated-p38 (p-p38), NOXA, BCL2, phosphorylated-BCL2 (p-BCL2). GAPDH as a loading control. **(P)** A375 cells were transfected with p38 siRNAs, and after 48 h transfection, the cells were treated with 5 μM of JW-1-283. After 24 h treatment of JW-1-283, cells were collected for immunoblotting analysis. P38, p53, p-p53, c-PARP and PARP protein expression were shown in the figure. GAPDH was used for loading control. **(Q)** A375 cells were transfected with p38 siRNAs, and after 48 h transfection, the cells were treated with 5 μM of JW-1-283. After 24 h treatment of JW-1-283, immunostaining for cyclin D1 (green fluorescence) was performed. Nuclei within each tumor were counterstained with DAPI (blue fluorescence). Scale bar = 50 μm. **(R)** JW-1-283 destabilizes the binding of MDM2 and p53, and therefore restored the function of p53. The stabilized p53 was phosphorylated by p38. The phosphorylated activated p53 could subsequently induced cell apoptosis as well as cell cycle arrest, which ultimately inhibits melanoma tumor growth. Figure was generated with BioRender.

Next, tumor behavior analysis was performed to investigate whether JW-1-283 could inhibit the aggressiveness of A375 cells. JW-1-283 significantly suppressed A375 cell migration in a dose dependent manner (Fig. 1C; Fig. S2C). Meanwhile, JW-1-283 significantly impaired the scratch healing (Fig. S2A, B). Since melanoma cells are highly likely to invade into the circulation and establish satellite colonies, we conducted cell invasion assay with A375 cell line using a Matrigel coated micropore membrane. As predicted, JW-1-283 significantly inhibited A375 cells to invade through the Matrigel matrix dose dependently (Fig. S2D, E).

Having confirmed the in vitro potency of JW-1-283, we investigated its anti-melanoma effect with a xenograft animal model. A375 melanoma cells were inoculated subcutaneously in the flank of NOD scid gamma (NSG) mice. Once the tumor has developed, animals were randomized into two groups to receive vehicle (saline) or JW-1-283 (7.5 mg/kg, i. p.) every other day. JW-1-283 significantly inhibited tumor growth with a total growth inhibition (TGI) value of 66% (Fig. 1D; Fig. S3A, B). Throughout the treatment, the animals did not show considerable weight loss or signs of abnormalities (Fig. S3C, D). Immunofluorescent staining was subsequently performed using the dissected tumor tissues. We observed a significantly reduced tumor proliferation and angiogenesis as indicated by Ki-67 and CD31, respectively (Fig. 1E). The treatment of JW-1-283 also enhanced the apoptosis with the tumor tissue as indicated by two well established markers, cleaved caspase-3 (cCas3), and cleaved poly (ADP-ribose) polymerase (cPARP) (Fig. 1E). We also examined the expression level of cyclin D1, which is one of the crucial cell cycle modulators that regulates cell phase. A significant inhibition on cyclin D1 was detected in the JW-1-283 treatment group, indicating our compound could potentially induce cell cycle arrest (Fig. 1E).

Considering the parent compound of JW-1-283 has demonstrated the capacity to impair the interaction of MDM2 and p53, we decided to investigate the potential action mechanisms of JW-1-283. To start with, we evaluated the protein stability of p53 and MDM2 with cycloheximide (CHX) pulse chase assay. When treated with JW-1-283, the stability of p53 has been improved whereas that of MDM2 was decreased (Fig. 1F). It may therefore imply that with the treatment of our compound, the interaction of MDM2 and p53 was interrupted, which could lead to the release of p53 to be activated.

It was reported that the phosphorylation of p53 at Ser15 is essential for its activation and subsequent induction of cell apoptosis.^4^ Therefore, we planned to examine the existence of Ser15 phosphorylated p53. With JW-1-283 treatment, the amount of p-p53 Ser15 within the A375 cell was significantly increased from 24 h and doubled after being treated for 48 h (Fig. 1G; Fig. S4A). Even though MDM2 expression was slightly increased, the protein level was not statistically changed (Fig. 1G; Fig. S4B, D), which might probably be associated with the treatment induced instability of MDM2. Next, the interaction between p53 and MDM2 was examined by co-immunoprecipitation assay. We found that JW-1-283 treatment could effectively decrease the amount of MDM2 bound to p53 followed by increased phosphorylation of p53 (Fig. 1H). Cell cycle arrest and apoptosis are the most noticeable biological outcomes of p53 activation. With JW-1-283 treatment, we observed significant apoptosis and S phase cell cycle arrest as indicated by Annexin-V apoptosis assay (Fig. 1I) and flow cytometry (Fig. 1J). In the meantime, cPARP protein expression showed increase both time and dose dependently (Fig. 1K, L; Fig. S5C, D) after treatment. Meanwhile, Cyclin D1 (G1/S cell phase regulator) protein expression also showed inhibition in both time and dose dependent manner (Fig. 1M, N; Fig. S5E, F).

p38-MAPK has been reported to play a dominant role in regulating p53 in the process of apoptosis.^5^ Therefore, we investigated the status of p38. In accordance with our prediction, the amount of the phosphorylated p38 was significantly increased with 24 h of JW-1-283 treatment in a concentration-dependent manner (Fig. 1O; Fig. S6A). Simultaneously, JW-1-283 treatment also increased the level of NOXA, which is a critical pro-apoptotic marker downstream the p38/p53 signaling (Fig. 1O; Fig. S6B). In addition, we observed that 5 μM of JW-1-283 treatment could reduce Bcl-2 phosphorylation at Ser70 significantly, which has been related with its antiapoptotic function (Fig. 1O; Fig. S6C).

To further verify that p38 is involved in the JW-1-283 induced p53 activation, we performed small-interfere RNA (siRNA) based gene knockdown targeting p38. Intriguingly, with the downregulation of p38, the amount of phosphorylated p53 was decreased compared with siRNA-control, even with the JW-1-283 treatment (Fig. 1P). Moreover, such a genetic interference also quenched the compound treatment induced apoptosis (Fig. 1P). Other than that, the genetic interference induced the expression of cyclin D1, which could be quenched by JW-1-283 treatment (Fig. 1Q). In summary, these results indicate that, by stabilizing p53, JW-1-283 could induce apoptosis and cell cycle arrest via the p38-MAPK pathway (Fig. 1R).

## Conflict of interests

The authors declare no conflict of interests.

## Funding

This work was partially supported by National Institutes of Health (10.13039/100000002NIH) grant R01CA240447 to W.L. Its contents are solely the responsibility of the authors and do not necessarily represent the official views of the NIH.
