# Supplementary material for: JW-1-283 inhibits melanoma tumor growth via stabilization of the p53 pathway
Source: Genes Dis. 2023 Jul 17;11(3):101036. doi: 10.1016/j.gendis.2023.06.009 (PMC10808915; doi:10.1016/j.gendis.2023.06.009)
Supplement: Multimedia component 1 [file mmc1.pdf]

Supplementary Information for

JW-1-283 inhibits melanoma tumor growth via stabilization of the p53 pathway

Yang Xie, Ruida Hou, Kelli L. Hartman, Jianxiong Jiang, Zhongzhi Wu[*], Wei Li[*]

Drug Discovery Center, College of Pharmacy, University of Tennessee Health Science Center, Memphis, TN, 38163, USA.

[*]Corresponding author:

Department of Pharmaceutical Sciences, College of Pharmacy, University of Tennessee Health Science Center. 881 Madison Ave, Room 561, Memphis, TN, 38163.

Email: wli@uthsc.edu, jimwu@uthsc.edu

This PDF includes:

Materials and Methods

Supplementary Scheme S1

Figures S1-S6

Material and methods

Cell lines and chemical compounds

The human melanoma cell lines (A375, M14, and RPMI7951) were purchased from American Type Culture Collection (ATCC). All the cell lines were cultured in Dulbecco`s Modified Eagle Medium (DMEM, Corning) supplemented with 10% fetal bovine serum (FBS, Atlanta Biologicals) and 1% antibiotic–antimycotic solution (Sigma-Aldrich) at 37 °C in a humidified atmosphere containing 5% CO_2. Cells were routinely tested for potential mycoplasma contamination using the MycoAlert Kit (Lonza) and were confirmed mycoplasma-free throughout the study. Compound JW-1-283 was synthesized in our lab. Cycloheximide for protein pulse chase assay was purchased from Cayman Chemicals.

Preparation of compound JW-1-283

The preparation of compound JW-1-283 was achieved from a two-step synthesis route (Scheme 1). Step 1. Synthesis of *N*-ethyl phenazinium ethylsulphate. Phenazine (1.00 g, 5.55 mmol) was dissolved in o-nitrotoluene (5 ml) at 140°C, then diethyl sulfate (1.71 g, 11.10 mmol) was added, and the resulting yellow solution was stirred for 1h at 145°C. The reaction solution was cooled in an ice bath and diethyl ether (50 ml) was added. The dark brownish solid was filtered and washed with diethyl ether to afford N-ethyl phenazinium ethylsulphate (1.65 g, 89% yield). HNMR (DMSO-*d6*): 8.94 (d, J = 8.4 Hz, 2H), 8.70(d, J = 8.4 Hz, 2H), 8.58-8.54 (m, 2H), 8.36-8.32 (m, 2H), 5.62 (q, J = 7.2Hz, 2H), 3.74 (q, J = 7.2 Hz, 2H), 1.71 (t, J = 7.2 Hz, 3H), 1.11 (t, J = 7.2 Hz, 3H). HRMS (ESI): calcd for $C_{14}H_{13}N_2$, 209.1079 $[M\text{-ethylsulfate}]^+$; found 209.0952.

Step 2. Synthesis of 2-(*N, N*-diethylamino)-9-Ethylphenaziniurn ethylsulphate (JW-1-283). N-ethyl phenazinium ethylsulphate (1.00 g, 2.99 mmol) was dissolved in methanol (10 ml) and diethylamine (0.26 g, 3.59 mmol) was added to the solution, which immediately turned dark violet. This solution was stirred for l hour at room temperature under air. Then the solvent was evaporated in *vacuo*. The residue was triturated with diethyl ether (3 x 10 ml), which was decanted. The residue was then subjected to silica gel chromatography ($MeOH/CH_2C1_2$ = 3/7 v/v) to give the desired compound as a purple color solid (0.85 g, 70%), LC purity, 99.0%. HNMR (DMSO-*d6*): 8.35-8.30(m, 2H), 8.13-8.10 (m, 2H), 8.02(d, J = 10 Hz, 1H), 7.87-7.84 (m, 1H), 6.79 (s, 1H), 5.00 (q, J = 6.4Hz, 2H), 3.92 (br, 4H), 3.74 (q, J = 7.2 Hz, 2H), 1.50 (t, J = 7.2 Hz, 3H), 1.32 (t, J = 6.4 Hz, 6H), 1.11 (t, J = 7.2 Hz, 3H). HRMS (ESI): calcd for $C_{18}H_{22}N_3$, 280.1814 $[M\text{-ethylsulfate}]^+$; found 280.1723.

Cell growth inhibition assay

Logarithmic growth phase cells were seeded in 96-well plates at a concentration of $3.5\text{-}5.0\times10^3$ cells/well depending on the growth rate of the cell line. After overnight incubation, the medium was replaced, and cells were treated with the test compounds at 10 concentrations ranging from 3 nM to 100 µM plus a vehicle (DMSO) control for 72 hours in three replicates. Following treatment, the MTS reagent (Promega, Madison, WI) was added to the cells and incubated in the dark at 37 °C for at least 1.5 hours. Absorbance at 490 nm was measured using a plate reader (BioTek Instruments Inc., Winooski, VT). IC50 values were calculated by nonlinear regression analysis using GraphPad Prism (GraphPad Software, San Diego, CA).

Sphere formation assay

For sphere formation assay, human A375 and M14 cells were seeded onto an ultra-low attachment 6-well plate (Corning) with a density of 8.0×10^3 cells/well. Tested compounds were added to the culture medium directly at concentrations indicated. Sphere formed by melanoma cells typically began to show 72 hours after seeding and continued being monitored under a microscope. At the end point of the treatment, the

spheres were analyzed, and diameters were measured using ImageJ software developed by the National Institutes of Health (NIH).

Colony formation assay

A375 cells and M14 cells were seeded into 12-well plates (5.0×10^2 cells/well, respectively) and then treated 48 hours later with increasing concentrations of JW-1-283. Medium was replaced with fresh drug twice per week. Cells were fixed with methanol and stained with 0.5% crystal violet, and colony morphology was visualized by a microscope and colonies quantified using ImageJ software (NIH, Bethesda, MD).

Cell migration and invasion assay

Cell migration and invasion were measured, respectively, using transwell 24-well plates with a noncoated membrane insert (pore size 8 µm). A375 cells were starved in serum-free medium for 24 hours, followed by suspension of the cells in serum-free medium containing 0.5, 1, 5 µM of JW-1-283 and then plating in the top chamber of the inserts. Medium containing 10% FBS was added to the lower chamber as a chemoattractant and cells were incubated for 24 hours. The chambers were fixed in cold methanol, stained with 0.5% crystal violet, and imaged. The number of migrating or invading cells was counted using ImageJ software and normalized to the control group for each cell line (as 100%). Random cell migration was then analyzed by a manual scratch wound healing assay. A375 cells (5.0×10^5 cells/well) were seeded in 12-well plates and allowed to adhere overnight. The following day, a scratch was made in the confluent cell monolayer using a sterile 200 mL pipette tip. The cell culture medium was replaced by medium containing 0.5, 1, 5 µM of JW-1-283.

In vivo tumor xenograft assay

All animal studies were performed under the guidelines of NIH Principles of Laboratory Animal Care and protocols approved by the UTHSC Institutional Animal Care and Use Committee (IACUC) (protocol #20-0166 for melanoma xenograft model and prostate xenograft model). Animals were housed under a 12:12 hours light/dark cycle at 20-26 °C with 30-70 % humidity. 8-9-week-old male humanized NOD scid gamma (NSG) mice (Jackson Laboratory) were used for implantation of A375 melanoma tumors. And 2.5×10^6 A375 logarithmically cells in 50 µL of medium were mixed with 50 µL Matrigel and subcutaneously injected into the dorsal right flank of each NSG mouse. A375 tumors were allowed to grow until the average tumor volume reached 100 mm^3. Tumor volume was measured using a caliper and calculated as $a \times b^2 \times 0.5$, where a and b represented the larger and smaller dimensions of the tumors, respectively. The mice were randomly assigned into 2 groups (n=7/group): vehicle (100% saline), 7.5 mg/kg of JW-1-283 with the same formulation for A375 xenograft model. Vehicle or JW-1-283 treatments were administrated intraperitoneally (i.p.) every other day for 16 consecutive days. During the treatment, tumor volume and body weight were recorded every 2-3 days. All mice were sacrificed at the conclusion of the treatment and tumors were collected, weighed, and photographed.

H&E and Immunofluorescence staining

Fixed organs were embedded in paraffin and stained with Hematoxylin eosin (H&E). Subcutaneous tumors were harvested and sectioned (10 µm) using an HM525 NX Cryostat (Thermo Scientific). The tumor tissue sections were fixed by 4% paraformaldehyde (PFA) at room temperature for 15 minutes and permeabilized with 0.25% Triton X-100 at room temperature for 10 minutes. After blocking in 10% goat serum in PBS at room temperature for 1 hour, the sections were incubated in primary antibodies at 4°C overnight: rabbit anti-Ki-67 (1:200, Biocare Medical, #CRM 325B); rat anti-cluster of differentiation 31

(CD31) (1:200, eBioscience, #14-0311-82); rabbit anti-cleaved caspase-3 (1:200, Cell Signaling Technology, #9664T); rabbit anti-cleaved-PARP (1:200, Cell Signaling Technology, #5625S), rabbit anti-cyclin D1 (1:200, Cell Signaling Technology, #55506S). The sections were washed with PBS and incubated with secondary antibodies: goat anti-rabbit IgG-Alexa Fluor 488 (1:1,000, Invitrogen, #A-11034), goat anti-rabbit IgG-Alexa Fluor 546 (1:1,000, Invitrogen, #A11035), at room temperature for 2 hours and DAPI (1 µg/mL in PBS) for 10 minutes. Stained sections were mounted on slides using DPX Mountant (Electron Microscopy Sciences). Images were obtained using a fluorescence microscope BZ-X800 (Keyence). The fluorescence intensity was quantified using ImageJ software (NIH).

Detection of apoptosis

A375 cells were seeded in 6-well plates (2×10^5/well). The next day, cells were treated with 5 µM of JW-1-283 for 24 hours. At harvest, cells from every well were suspended in 185 µL of Annexin-V-FITC binding buffer (eBioscience) and 5 µL of Annexin-V-FITC (eBioscience) and 10 µL propidium iodide (PI) were added, followed by incubation for 10 minutes at room temperature, and analysis in the UTHSC Flow Cytometry and Cell Sorting core.

Cell-cycle analysis

After treatment, cells were harvested by trypsinization, fixed, permeabilized, and then incubated with freshly prepared propidium iodide/RNase solution for 30 minutes at room temperature. Data were analyzed using FlowJo version 10 (FlowJo, LLC) in the University of Tennessee Health Science Center (UTHSC) Flow Cytometry and Cell Sorting core. First, total cells were gated, followed by gating out cell doublets and debris. Cells in G1, S, and G2/M phases were then gated according to DNA content, as determined from histogram plots of PI staining-Area.

siRNA transfection

A375 cells were seeded into 6 well plates at a density of 2.5×10^5 cells/plate and allowed to reach the confluency of 80%. A375 cells were transfected with siRNA p38 or a scrambled RNA (control) (#6564S, #6243S, #6568S Cell Signaling Technology, Inc) at a final concentration of 100 nM using Lipofectamine 3000 according to the manufacturer's instructions. After 48 hours, replace medium containing 5 µM JW-1-283. After incubation for 24 hours, cells were harvested for analysis.

Western blot

A375 cells were seeded into 6 well plates at a density of 2.5×10^5 cells/plate and allowed to attach overnight. The medium was replaced with either fresh complete DMEM medium or DMEM containing the desired concentration (0.25, 0.5, 1, 2.5, 5 and 10 µM) of JW-1-283. After 24 hours of incubation, cells were washed with PBS, lysed with RIPA buffer (Thermo Fisher Scientific) containing halt protease and phosphatase inhibitor (Thermo Fisher Scientific), and centrifuged at 13,000 rpm at 4 °C for 15 minutes. The BCA method was used to quantify the protein in each sample. 30 µg of each sample were subjected to western blot analysis as described. The protein samples were separated via SDS-PAGE, transferred to PVDF membranes, blocked with 5 % nonfat milk for 1hour at room temperature followed by overnight incubation at 4 °C with primary antibodies, which included mouse anti-p53 antibody #48848S, rabbit anti MDM2 antibody #86934S, rabbit anti-GAPDH #3683S, rabbit anti-phospho-p53 antibody 9284S, rabbit anti-cleaved-PARP antibody #5625S, rabbit anti-PARP antibody #9532S, rabbit anti-cyclin D1 antibody #2978S, rabbit anti-β tubulin antibody #5568S, rabbit anti-p38 antibody #8690T, rabbit anti phospho-p38 antibody #4511T, rabbit anti-phospho-BCL2 antibody #2827T (Cell Signaling Technology, Inc), rabbit anti-NOXA antibody #FL-54 and mouse anti-BCL2 antibody #SC-7382 (Santa Cruz Biotechnology, Inc).

Primary antibodies were detected with HRP-conjugated secondary antibodies (#7074, #7076 Cell Signaling Technology, Inc). And immunoreactive bands were visualized using enhanced chemiluminescent substrate (ECL, Thermo Fisher Scientific).

Statistical analysis

All data were analyzed using GraphPad Prism 9.0. *In vitro* experiments were repeated using at least three technical replicates per group and each assay was performed over three biological replicates. One-way or two-way ANOVA tests were first employed for experiments comparing more than two groups/conditions, followed by a Dunnett multiple comparison test. Significance levels are defined as, $*P < 0.05$; $**P < 0.01$; $***P < 0.001$; $****P < 0.0001$.

Supplementary Scheme S1. Synthesis of MX25 and JW-1-283

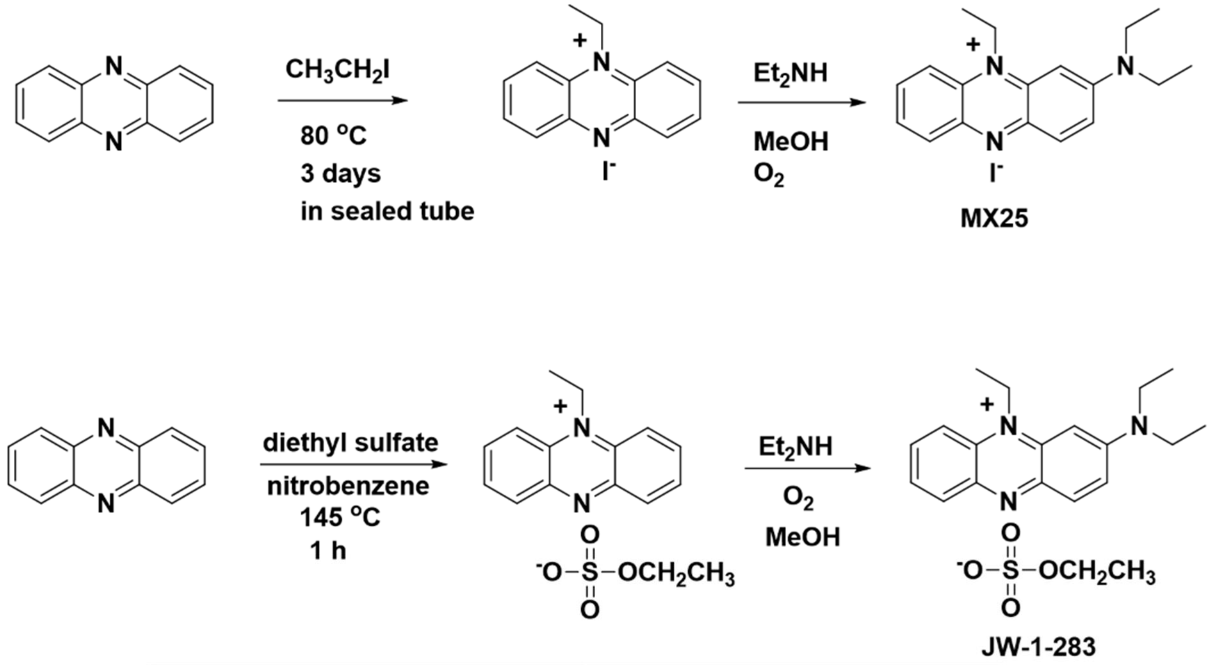

Compound	IC50 (μM) (mean±SEM)
MX25	6.3±1.0
JW-1-283	3.1±0.7

Note: Compound potency was tested in the A375 cell line

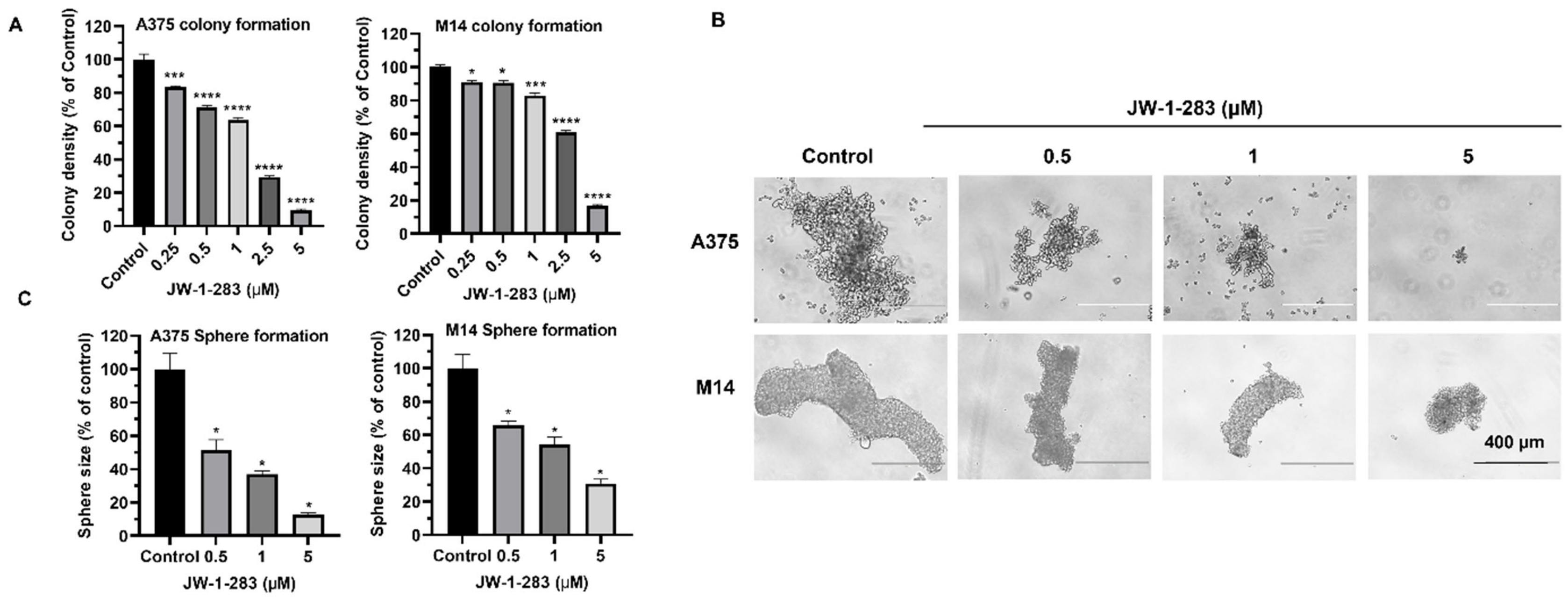

Figure S1. JW-1-283 inhibits human melanoma cell proliferation, especially in p53 WT melanoma cell line. (A) Quantification of colony formation area is expressed as the grand mean ± SEM compared with vehicle control (set to 100%) of three biological replicate experiments. **(B)** Effects of indicated concentration of JW-1-283 from 0.5 μM to 5 μM on A375 and M14 spheroids, for up to 8 days of treatment. **(C)** Quantification of sphere formation is expressed as the grand mean ± SEM compared with vehicle control (set to 100%) of three biological replicate experiments.

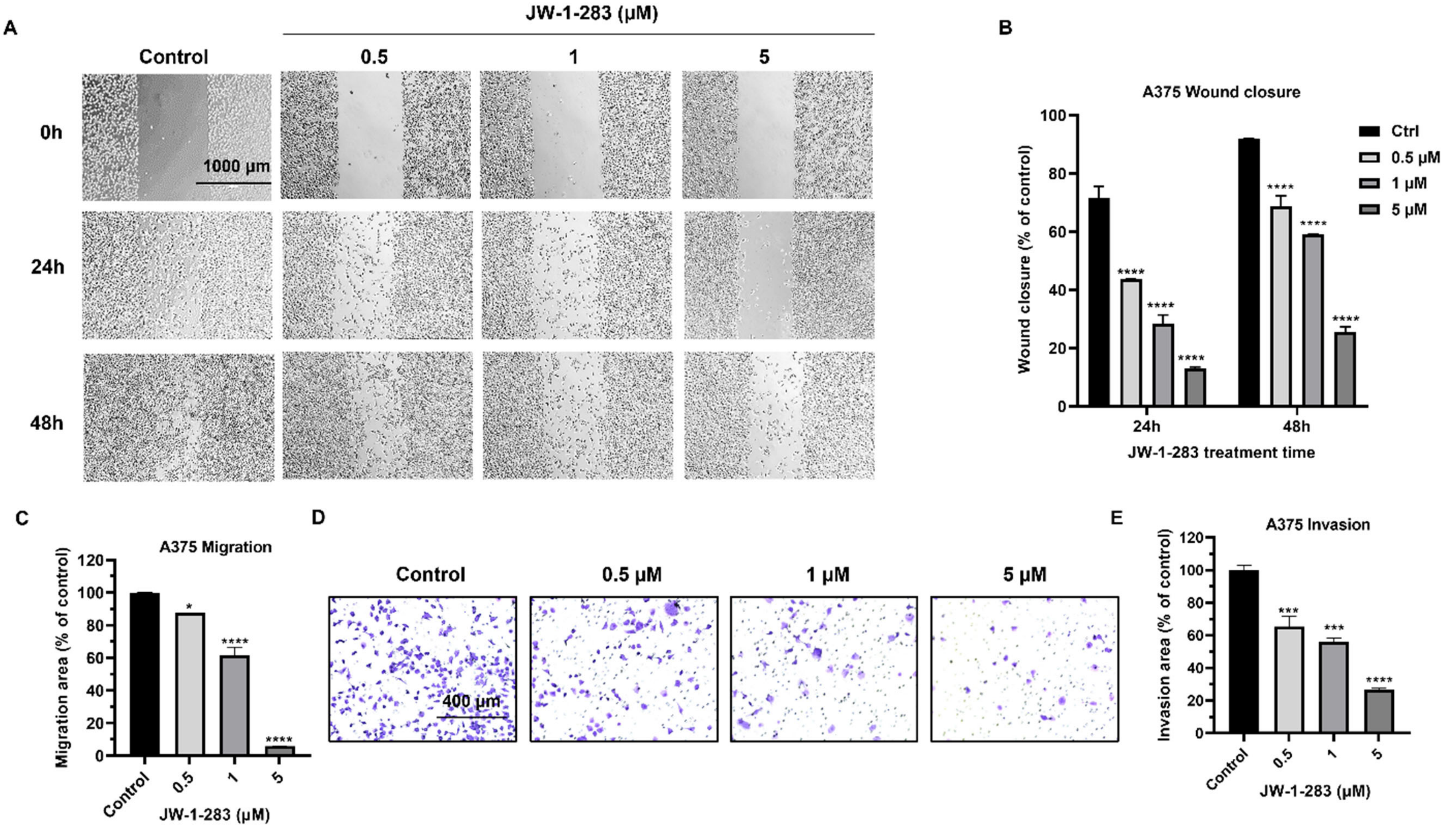

Figure S2. JW-1-283 reduces melanoma wound closure, cell migration as well as invasion. (A) The effect of JW-1-283 on A375 cells on wound healing was determined by the scratch assay after treatment for 24 and 48 hours, respectively. Scale bar=1000 µm. (**B)** Quantification of wound healing closure rates. A375 melanoma cells treated with JW-1-283 were determined by the percentage of wound closure relative to cells in the control group. **(C)** Quantification of cell migration area is expressed as the grand mean ± SEM compared with vehicle control (set to 100%) of three biological replicate experiments. **(D)** Directed invasion of A375 cells was determined using a transwell 24-well plate following treatment with JW-1-283 from 0.5-5 µM at 24 hours using crystal violet staining. Scale bar= 400 µm. **(E)** Quantification of cell migration area is expressed as the grand mean ± SEM compared with vehicle control (set to 100%) of three biological replicate experiments.

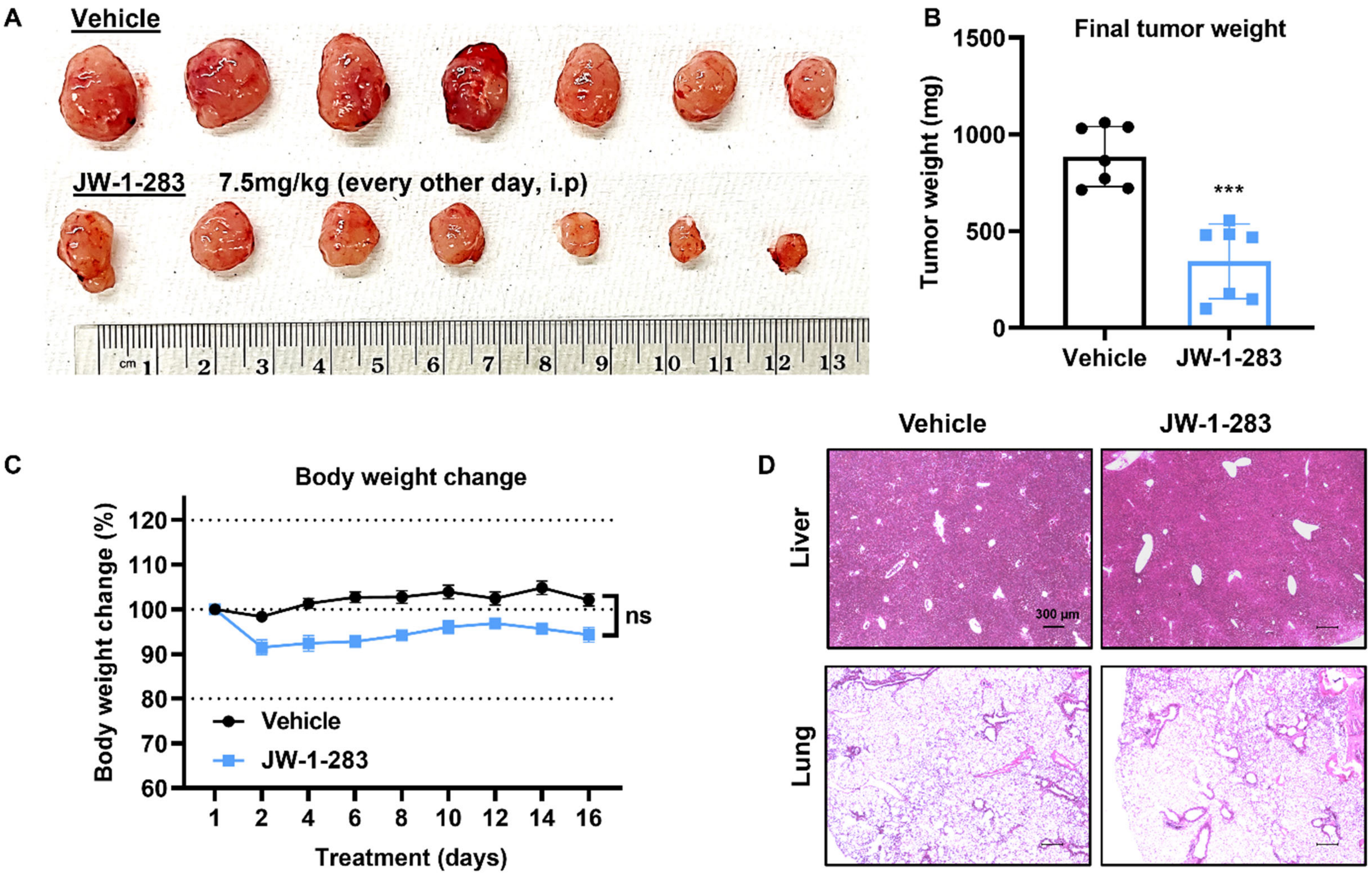

Figure S3. JW-1-283 suppresses melanoma tumor growth *in vivo.* (A) . Representative images of tumors from A375 cells xenograft NSG mice with the intraperitoneal injection of 7.5 mg/kg JW-1-283 or saline. (**B)** Quantification of the average tumor weight at the end of the treatment. **(C)** Mean percent change in mice body weight ± SEM relative to body weight at the initial treatment time. Mice body weight were recorded every other day. **(D)** HE staining of liver and lung from the JW-1-283 treated mice and vehicle group. Scale bar=300 µm.

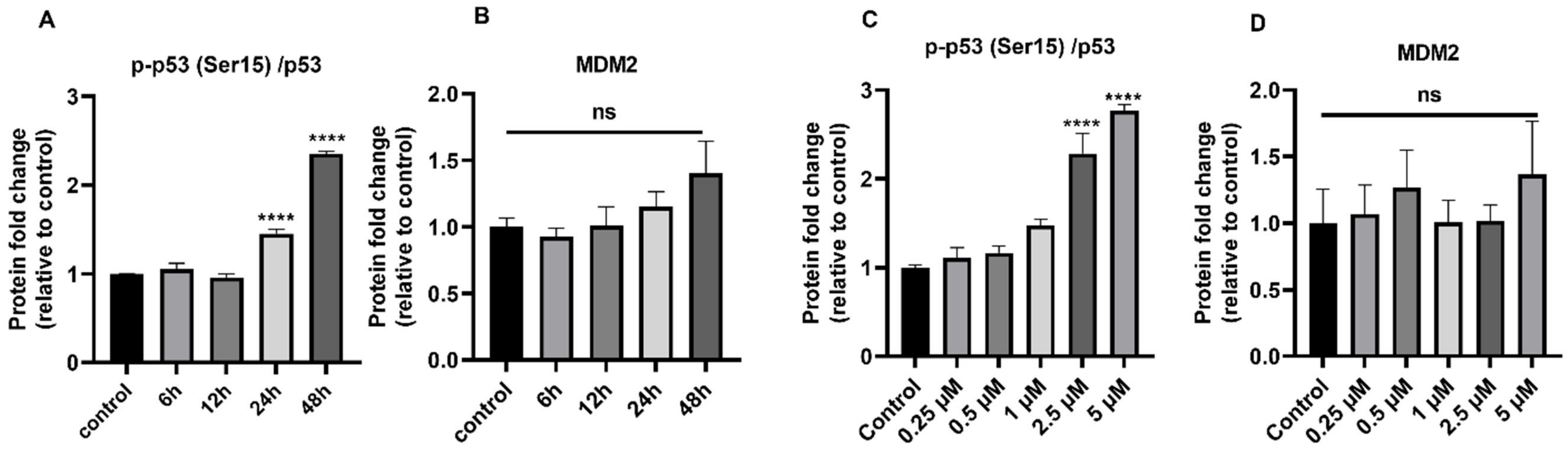

Figure S4. JW-1-283 destabilizes the interaction of MDM2 and p53. (A) and (B) Quantification of the relative fold change of p-p53/p53, MDM2 compared with vehicle control (set to 1) in the time dependent experiment. **(C) and (D)** Quantification of the relative fold change of p-p53/p53, MDM2 compared with vehicle control (set to 1) in the dose dependent experiment.

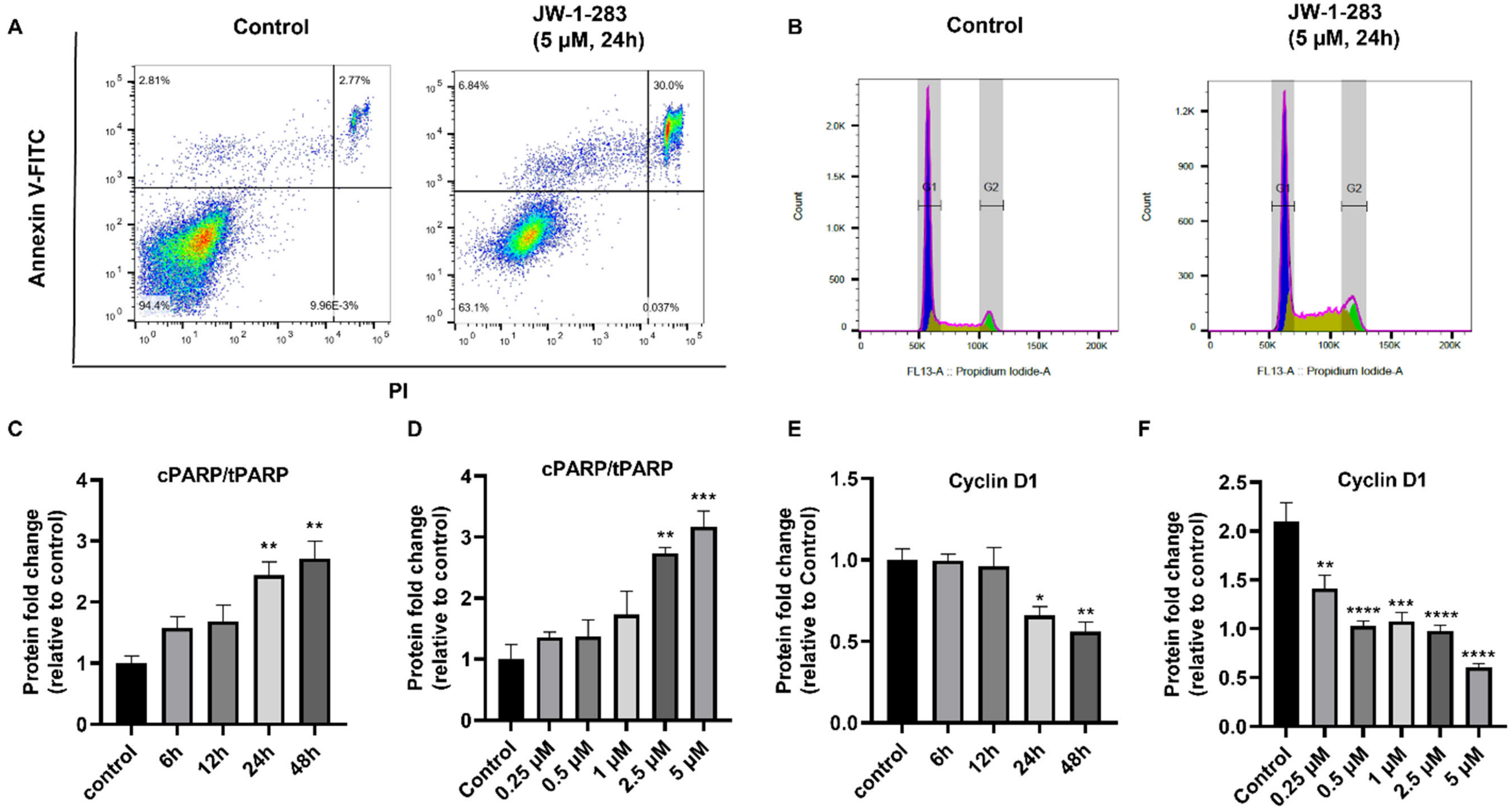

Figure S5. JW-1-283 induces apoptosis and cell cycle arrest in a time- and dose- dependent manner. (A) Apoptosis was compared after treating with 5 µM of JW-1-283 for 24 hours by Annexin-V/PI co-staining. **(B)** A375 cells were treated with 5 µM of JW-1-283 after 24 hours. Cell-cycle distribution was determined by flow cytometry after staining with PI. **(C) and (D)** Quantification of the relative fold change of cPARP/total PARP compared with vehicle control (set to 100%) in the time and dose dependent experiment. **(E) and (F)** Quantification of the relative fold change of Cyclin D1 compared with vehicle control (set to 1) in the time and dose dependent experiment.

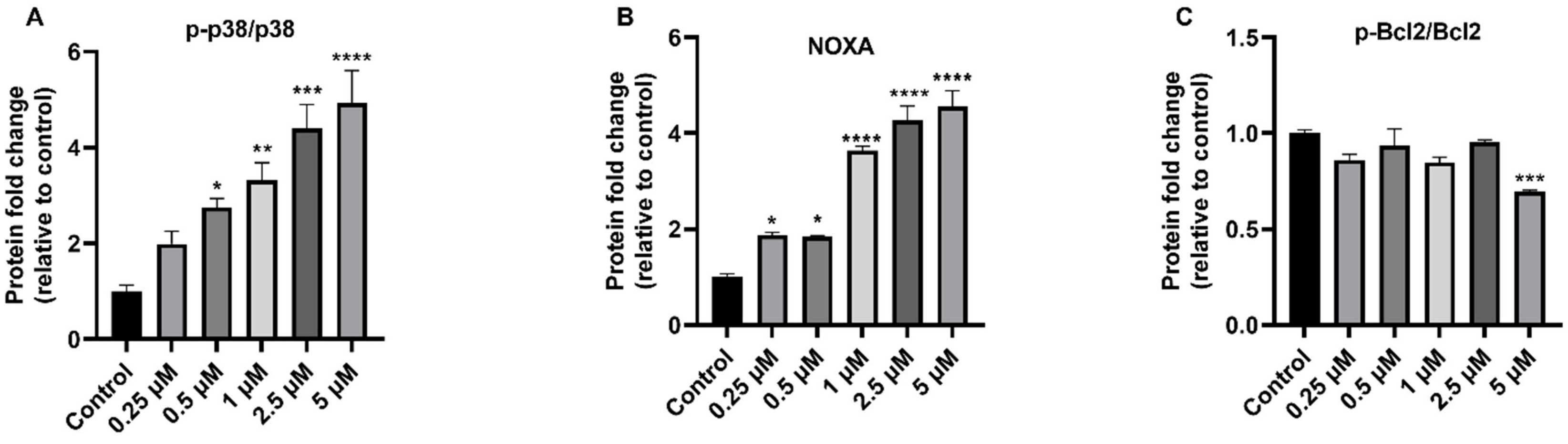

Figure S6. JW-1-283 stabilizes p53 is phosphorylated by p38. (A), (B) and (C) Quantification of the relative fold change of p-p38/p38, NOXA, p-BCL2/BCL2 compared with vehicle control (set to 1) in the dose dependent experiment.
